# Burnout among Social Workers in Iran: Relations to Individual Characteristics and Client Violence

**DOI:** 10.5539/gjhs.v5n4p142

**Published:** 2013-05-02

**Authors:** Mojgan Padyab, Jörg Richter, Lennart Nygren, Mehdi Ghazinour

**Affiliations:** 1 Department of Social Work, Umeå University, Umeå, Sweden; 2 Centre for Children and Adolescents Mental Health, Oslo, Norway

**Keywords:** burnout, social work, self-esteem, client violence, Iran

## Abstract

Social workers are considered a professional group at high risk of burnout. Noticing the insufficient human resource management and understaffed social work centers, Iranian social workers are faced with a considerable level of physical and mental stress, which can lead to burnout. A national study on 390 social workers was conducted. Among social workers, 10.9% had experienced burnout and 17.4% are at risk of developing burnout. Social workers scored higher in burnout if they were dissatisfied with their income, had experienced violence, or had lower self-esteem. Findings are discussed with regard to Iranian context and recommendations for authorities of Iranian state welfare organizations are made.

## 1. Introduction

Ever since the 1970s, the interest in burnout among researchers, practitioners and general public has been dramatically increased almost everywhere around the globe (Schaufeli et al., 2009). The significance of burnout lies in its correlation with important outcomes such as the quality of professional work and practice conduct; productivity; and health related issues (Maslach et al., 2001). Descriptions and discussions around burnout began in the mid-1970s concerning helping professions and occupations that involve intense human interactions, such as health care, social work and teaching. Social work burnout has received great research attention as social workers constantly face limited resources while meeting demanding clients that require immediate attention (Söderfeldt et al., 1995). One of the main contributing factors to burnout is the constant imbalance of demands over resources (Bakker & Demerouti, 2007), and therefore social work represents a prime example of burnout.

Social workers are considered a professional group at high risk of burnout (Jayaratne & Chess, 1984). Conflict is more likely to arise because social work is strongly client related and practitioners are involved in complex situations. Social workers’ burnout has become a crucial managerial issue because of its negative impacts on quality and consistency of client services (Kim & Lee, 2009); and its association with job dissatisfaction, which results in leaving the job or professional misconduct (Jayaratne & Chess, 1984; Kim & Stoner, 2008; Reamer, 2006). It is known that burnout level and its contributors differ across nations (Schaufeli & Enzmann, 1998). Discussion about nations must acknowledge the differences in work environment, demographic, work-related variables and cultures (Savicki, 2002). There is a limited number of studies on burnout in Iran and most of them focused on other occupations such as nurses, psychologists and occupational therapists (Fazelzadeh et al., 2008; Ashtari et al., 2009; Nayeri et al., 2009). The results from burnout among Iranian nurses (Nayeri et al., 2009) revealed that burnout was associated with lower productivity and they tend to evaluate themselves negatively, especially regarding their work with other people. The situation is not all that different among social workers as they are also considered a “helping profession”. Noticing the very slow increase in the number of social workers in the last few years and understaffed social work centers (Iranian Social Work Association, 2010), Iranian social workers are faced with a high level of physical and mental stress (Author, 2011). The official statistics from Iranian Social Work Association shows that there were approximately 3000 social workers in Iran in 2006, or 1 per 23,000 population and it reached to 1 per 7,500 population in 2012. As a young profession, it may still have a long way before it is recognized as a profession dedicated to deal with vulnerable clients. Iranian social workers are faced with an increasing number of problems in their daily work and yet, no investigation is available on their work related health conditions including burnout.

### 1.1 Predictors of Burnout

Previous investigations have shown that burnout is, in fact, multidimensional and various variables, such as clients’ character, workers’ character, and work setting are related to burnout (Maslach et al., 2001). The diversity of antecedents of social work burnout includes job-related variables, personality dimensions and individual conditions. Most of the researchers argue that social and work-related variables (e.g., demanding job conditions and supervisory communication) are stronger predictors of burnout than individual variables (Söderfeldt et al., 1995). Likewise, Maslach and colleagues (2001) argue that burnout is mainly related to the work context such as role conflict, ambiguity, lack of resources and supervisory. However, the findings suggest that demographic/individual variables are related to higher susceptibility of burnout. Burnout among practitioners and workers in human services decreases as they grow older (Francis et al., 2009). Female workers tend to be emotionally involved and dedicated, and therefore, they experience less depersonalization than do male workers (Erickson & Ritter, 2001; Kulik, 2006). There is evidence that single individuals report higher levels of burnout because of less familial support and low income is related to higher susceptibility to burnout (Cordes & Dougherty, 1993). Furthermore, social workers who have experienced client violence develop feelings of helplessness and demoralization (Spencer & Munch, 2003). Abdallah (2009) reported that burnout is negatively related to self-esteem. Self-esteem refers the extent to which one prizes, values, approves, or likes oneself or simply how positively or negatively we feel about ourselves (Passer & Smith, 2008).

### 1.2 Aim

Even though there is a shortage of knowledge on the burnout level and its predictors among Iranian social workers, this has not yet led to systematic investigations. As a primary step to bridge the knowledge gap in this topic, the present study focuses on the individual social worker with the aim to examine how burnout is associated with age, gender, satisfaction with income, civil status, experience of client violence, and self-esteem. The choice of variables associated with burnout could be made considering organization, client and individual social worker. Most burnout research targets the professional as individual and that our choice is partly reflecting this strand of research in order to make future comparisons possible. Besides, social workers play a key role to implement social interventions in Iranian society and their individual well-being is crucially important.

## 2. Methods

The presented findings are part of a more comprehensive national investigation on mental health among Iranian social workers. The research was conducted in collaboration with the State Welfare Organization, Iran Medical University of Tehran and Department of Social Work at Umeå University in Sweden. The research proposal was approved by Research department of the State Welfare Organization in Tehran and the ethics committee in Iran Medical University of Tehran.

### 2.1 Sample

The current study was conducted in all 30 provinces of Iran between 1 July and 15 September 2009. There are Centers for Socially Injured People (CSIP) affiliated to Social Affairs Department of the State Welfare Organization in cities of all provinces. These centers have approximately 500 staff. From all centers a total number of 390 social workers who were present at work during the data collection period were included in the study. The number of CSIP social workers in our sample ranged from 76 social workers from Tehran, the most populated province in Iran with 11,305,832 urban inhabitants (Statistical Center of Iran, 2011) to 6 CSIP social workers from Kohgiluyeh and Boyer-Ahmad, the least populated province in south-west of Iran with 346,626 urban inhabitants (Statistical Center of Iran, 2011).

### 2.2 Setting

The current study concentrates on CSIPs. Social workers in these centers are involved in activities such as family crisis interventions and social emergency telephone line (123). The interested reader can find details about these centers elsewhere (Author, 2011). These centers are involved in a diverse range of social interventions and are considered as one of the important organizations in social work practice in Iran. The clients of CSIPs are mainly homeless people, abused children, drug users, street children and labor children. CSIP Social workers are very likely to face clients with uneasy issues and mostly seek for immediate attention and therefore it often involves inevitable challenges, risks and tension.

### 2.3 Instruments

The survey included a sociodemographic form and a self-administered questionnaire in reference to violence at work, general mental health, burnout and self-esteem. Social workers were informed about the purpose of the study and volunteer participation was ensured by a written consent form.

*Workplace violence* was assessed using the “Workplace Violence in the Health Sector” questionnaire (ILO/ICN/WHO/PSI, 2003). The definition of violence, either physical or psychological, was given to the participants. Physical violence is defined as using physical force against another person, including beating, kicking, slapping, pushing, shooting, stabbing, biting, and pinching. Psychological violence is defined as intentional use of power including the threat of physical force against another person that can cause harm to mental, spiritual or social development. It includes verbal abuse, bullying/mobbing, harassment, and threat.

The questionnaire was originally in English and then translated to Farsi (the official language in Iran). We distributed the Farsi version of the questionnaire to 25 staff members of the State Welfare Organization in Tehran and made necessary changes to make sure about the face validity.

*General Mental Health* was measures by the General Health Questionnaire (GHQ-28). This screening instrument was developed to detect minor psychological disturbances in a community sample and nonpsychiatric clinical settings (Goldberg & William, 1988). The GHQ-28 is validated in Iranian setting (Noorbala et al., 1999) and is used to assess the general mental health by four scales of physical symptoms, anxiety and sleep disorders, social dysfunction, and depression. The scoring is based on the 0-0-1-1 method, and the higher the score, the poorer the mental health status. The best cutoff point to detect possible cases of mental disorder is 6 (Noorbala et al., 1999). Cronbach's alpha coefficient for GHQ-28 items was 0.92 in our data.

*Burnout* was assessed by the Burnout Measure (BM) (Pines et al., 1981). The 21 items in this instrument correspond to the three components of the burnout, including physical exhaustion (for example, “being tired” and “feeling weak”), emotional exhaustion (for example, “feeling burned out” and “feeling depressed”), and mental exhaustion (for example, “being unhappy” and “feeling rejected”). The response to all items is on a seven-point Likert scale from one (*never*) to seven (*always*), and a higher score reflects more severe symptoms of burnout. The mean value of responses to all 21 items (with positive items reversed) is considered as the burnout score. Occasionally, proposed norms (Pines et al., 1981) are used to classify people who experience different levels of burnout as follows: no burnout (≤2.9), at risk of developing burnout (3-3.9), and experienced burnout (4 and higher). The BM is highly correlated with the emotional exhaustion subscale (the only intrinsic dimension of burnout) of the Maslach Burnout Inventory (Schaufeli & Van Dierendonck, 1993). The Farsi version of BM was developed following established guidelines (Sartorius & Kuyken, 1994). This procedure includes translation, back translation, pilot testing, and determining the semantic equivalence of the translation.

*Self-esteem* was measured using Rosenberg Self-esteem Scale. Rosenberg Self-esteem Scale contains 10 items, half of which are positive (e.g., I feel that I have a number of good qualities), and the other half are negatively worded and reversed scored (e.g., I feel I do not have much to be proud of). Responses vary from 1 (*strongly disagree*) to 4 (s*trongly agree*). The total score is the sum of 10 items (possible range 10 to 40) and a higher score on the scale indicates a higher self-esteem. The internal consistency of the scale and concurrent and construct validity of the instrument are tested in an Iranian sample (Shapurian et al., 1987). In our study, Cronbach's alpha coefficient was 0.82.

### 2.4 Statistical Methods

Factor analysis using principle axis factoring and oblique rotation was performed to test the factor structure of the BM instrument. Parallel analysis (Hayton et al., 2004) and scree test were used to ascertain the number of factors. Cronbach's alpha scores were calculated to ascertain reliability measures. In the comparisons of burnout score between groups, we used burnout score measured at interval scale (continuous values) as dependent variable. Independent variables were gender, age, satisfaction with income, civil status, experience of violence and self-esteem. We applied t-tests for independent samples to search for differences of burnout between groups, Pearson correlation coefficient to identify relationships between continuous variables, and analysis of covariance (ANCOVA) to test if change in burnout score depends on levels of satisfaction with income, experience of violence, and self-esteem, holding all else constant. Self-esteem was considered as the covariate because it was the only variable with interval scale (continuous values) in the model. Satisfaction with income and experience of violence were both categorical and therefore were considered as fixed factors.

## 3. Results

### 3.1 Dimensionality and Validity of the Burnout Measure

Based on the result from exploratory factor analysis, the optimal number of factors to be retained proved to be three, which explained 67% of variance. The factors assumed by Pines and Aronson (1981) were Physical exhaustion (items 1, 4, 7, 10, 13, 16, 20); emotional exhaustion (items 2, 5, 8, 12, 14, 17, 21); and mental exhaustion (items 3, 6, 9, 11, 15, 18, 19). None of the factors assumed by Pines and Aronson (1981) emerged and items belonging to these factors were too spread across factors in our data set. Based on the results from our analysis, the three factors thus can be labeled as: *demoralization* (items 7, 10, 11, 12, 14, 15, 16, 17, 18, and 21), *exhaustion* (items 1, 2, 4, 5, 8, 9, and 13), and *loss of motive* (items 3, 6, 19, and 20). The factor correlations were rather high (range: 0.6 to 0.63). The reliability of all three factors as well as the total score is high (demoralization: α = 0.93, exhaustion: α = 0.92, loss of motive: α = 0.82, and total BM score: α = 0.96). Item 8 (“Feeling burned out”), which is supposed to be the best indicator of burnout in BM, was highly correlated with total score excluding item 8 (r = 0.85, P < 0.001). Burnout score ranged from 1 to 5.8, and the 75^th^ percentile was found to be 3.2 in this study.

A high correlation was found between burnout score and GHQ total score (r = 0.71, p < 0.001). Since higher GHQ scores indicate decreased mental health status, this finding suggests that burnout is positively correlated with poor health and indicates convergent validity because burnout is expected to be positively correlated to poorer health (Schaufeli & Enzmann, 1998). In addition, 86% of social workers who experienced burnout (burnout score four and higher) fell into the group who have GHQ scores of six or higher (the cut point of classification of possible cases of mental disorder) (Noorbala et al., 1999).

### 3.2 Comparison of Burnout Score between Groups

The demographic variables of social workers, who participated in the study, as well as their self-esteem, burnout score, and experience of violence, are presented in Table 1. The results show that 10.9% of social workers had experienced burnout and 17.4% are at risk of developing burnout.

**Table 1:** Demographic characteristics of social workers and their self-esteem, burnout score, and experience of violence (n = 390)

	%	Mean (SD)
Gender		
Male	16	
Female	84	
Age (yrs)		32 (6.3)
<25	8.6	
25-29	32.4	
30-35	33.6	
36-39	14.4	
≥40	11.0	
Satisfaction with income		
No	56	
Yes	44	
Civil status		
Single	33.6	
Married	66.4	
Experience of violence in the last 12 months		
None	32.3	
Physical	3.0	
Verbal	44.7	
Both	20.0	
Self-esteem		33 (4.7)
Burnout Measure (BM)		2.6 (1.0)
No burnout	71.7	
Risk of developing burnout	17.4	
Experienced burnout	10.9	

We examined whether social workers’ burnout scores differed relative to their gender, age, satisfaction with their income, civil status, experience of violence, and self-esteem. Male and female social workers showed no difference with regard to burnout (2.4 ± 0.96 vs. 2.6 ± 1.01, respectively). T-test revealed that the burnout score for those who were dissatisfied with their income was significantly higher than those who were satisfied (2.7 ± 0.9 vs. 2.4 ± 0.9, respectively; p < 0.01). No difference was found between single and married workers in levels of burnout. Social workers with experience of violence of any kind were found to have a higher burnout score compared with those without experience of violence (2.7 ± 1.0 vs. 2.3 ± 0.9, p < 0.001, respectively). The burnout score was significantly negatively correlated with self-esteem (r = -0.61, p < 0.001) but not with age (r = -0.08, p = 0.13). Burnout was significantly associated with experience of violence, satisfaction with income and self-esteem. These variables were simultaneously considered as independent variables in ANCOVA analysis and the main effect of each (separate contribution to mean burnout score, holding all else constant) was calculated. The results showed significant main effects of self-esteem (p < 0.001; F_(1,264)_ = 152.4; η^2^ = 0.37) and experience of violence (p = 0.004; F_(1,264)_ = 8.6; η^2^ = 0.03) based on burnout score and non-significant main effect of satisfaction with income (p = 0.16; F_(1,264)_ = 2.0; η^2^ = 0.008).

## 4. Discussion

In this study, the factorial structure of the BM that has been found by Enzmann, Schaufeli, Janssen, and Rozeman (1998) was replicated. Instead of the hypothesized factors (“emotional exhaustion,” “physical exhaustion,” and “mental exhaustion”), three other factors (“demoralization,” “exhaustion,” and “loss of motive”) emerged. The high correlation between factors justifies the calculation of one single burnout score. Because neither a clinically valid nor a statistically valid criterion for burnout exists, we do not know how many social workers are actually “burned out” and the prevalence cannot be assessed in absolute terms. Previous research has shown that BM's discriminant validity is unsatisfactory; ‘exhaustion’ and ‘demoralization’ can hardly be differentiated from psychosomatic symptoms (Enzmann et al., 1998). This result raises questions with regard to whether there is a statistically valid instrument to measure burnout. On the other hand, from clinical perspective, no burnout diagnosis (clinically validated cut-offs) are defined and all clinical forms of burnout are best covered by the existing ICD-10 diagnosis guidelines for Neurasthenia, if work related. Therefore, we report the relative occurrence of burnout symptoms throughout this paper.

The findings of our study did not support the view that some social workers with different demographic/individual differences (age, civil status, and gender) are particularly susceptible to burnout. This is in line with Maslach's (1998) statement that no key individual predictors have emerged, although there has been a long-standing interest to identify such variables. The absence of definite association between burnout and personal characteristics may be due to lack of relevant theory and conceptual model for hypothesizing which individual variables should be most predictive and why (Maslach, 1998). Associations between burnout and demographic variables tend to diverge but there may be some consistency in that persons who are unmarried and at younger age seem to be more prone to burnout (Maslach et al, 2001) and data about gender differences in burnout are unconvincing. The participants of our study were relatively homogenous with regard to these demographic characteristics, which limit the variation to show possible differences.

Our findings corroborate the importance of self-esteem, satisfaction with income and experience of violence on burnout. The correlation between financial strain and income level with burnout has previously been addressed (Soares et al., 2007). However, income variable is no longer significant in our study after controlling for experience of violence and self-esteem.

It was found that BM score was higher among those with experience of violence. Burnout is a particular feature of chronic and prolonged stress (Collings & Murray, 1996; Lloyd et al., 2002). Stress is defined as a person-environment interaction that is appraised by the person as taxing or exceeding his or her resources and as endangering his or her well-being (Folkman, 1984). Although there are difficulties of assessing whether the stress is due to work, home, or a combination of the two, Gibson, McGrath and Reid (1989) suggested that social workers perceive their work as the source of substantially more stress than their personal life. The very core of social work lies in relationship with clients, and for most social workers, sensitivity to client's problems makes them vulnerable to work stress (Maslach, 1998). Exposure to violence is undoubtedly one of the stressful triggers, which is associated with altered biological stress-adaptation systems (Crofford, 2007), especially when occurring combined with a lack of leader's support, and sustained alteration of stress-adaptation systems may result in ill health and burnout. Newhill (2003) argues that violence affects how social workers approach their clients. Social workers in her study reported that, following a violent incident, they were more guarded and wary around clients, keeping clients at a distance; were less empathic toward clients; and becoming less involved with clients emotionally as a means of self-protection. It is noteworthy that 67% of social workers in our study experienced violence of some kind with higher magnitude of psychological violence than physical violence. It is difficult, if not impossible, for these social workers to remain unchanged to information that bears on their self-esteem, such as being told that they are incompetent, untrustworthy, or incapable.

Instead of a personality trait that may predispose individuals to burnout, poor self-esteem also could be caused by environmental factors or even by being burnt out (Schaufeli & Enzmann, 1998). The only longitudinal study that investigated the causal relationship of self-esteem and burnout found no conclusive evidence about their causal order (Rosse et al., 1991). The negative correlation between self-esteem and burnout found in our study is consistent with other studies (Abdallah, 2009; Rosse et al., 1991). However, it is unclear whether low self-esteem is an antecedent or consequence or burnout. Rather, we emphasize on the conclusion made by Rosse et al. (1991) that self-esteem may be an important factor in predicting who will be more likely to develop burnout. The research has been generally consistent with the notion that the core characteristics of people with high self-esteem is their tendency to persist in the face of failure and the ability of utilization of more adaptive self-regulatory strategies (Baumeister et al., 2003). In fact, many social workers tend to blame themselves when an incident occurs. That may be due to the fact that social work training/education emphasize that prevention of violence is a task requiring to be handled with proficiency (Chelak, 2009). When violence happens, often the social worker's attitude is that this happens because they did not possess adequate professional expertise. This feeling of self-blame, shame, and incapability may undermine their sense of competence, self-worth, and diminish their self-esteem. It is crucial for social workers not to let their self-esteem be affected by the notions of “worthless” or “useless” or any other kind of verbal abuse or harassment from vicious clients.

In the current study, BM was used, which was originally designed for use with all occupational as well as non-occupational groups. The BM is not restricted to ‘people work’ and it is assumed that burnout can occur in any occupational field as well as in other groups such as couples, parents and housewives (Enzmann et al., 1998). Our intention for using BM instead of other instruments, which are designed specifically for human service professions, was that we believe what a social worker encounters at work does not end there and the burden goes further to their family and private life. Eighty four percent of the study social workers are women and for many Iranian women, traditional culture norms and their roles as mothers and homemakers are intrinsically valued.

We found that 17.4% of social workers are at risk of developing burnout and 10.9% have already experienced burnout. These figures tell us that nearly one third of Iranian social workers are suffering or will suffer from burnout. With regard to negative impacts of burnout on individuals’ health as well as quality of practice, it calls for immediate attention and care from authorities of social work organizations. If we assume that burnout is more likely to occur among social workers with different individual and psychosocial work characteristics (risk factors) what would such associations imply in terms of policy? It is clear that the effect of such risk factors on burnout would reduce via interventions that target the inequality of these factors. As the first study among social workers in Iran, we investigated some potential contributing factors to burnout which might be translated into policy. Violence and self-esteem were found significant risk factors and therefore Iranian social work authorities are asked to think carefully about them in order to reduce their impact on social workers’ burnout. The CSIP social workers are considered as first contact for many different needy people such as those with mental health issues, domestic violence, and couples in divorce crisis. Knowing about such problems could help the reader to visualize the image of difficulties that Iranian social workers face in their daily work, and explain the reason that 10.9% of them had experienced burnout and 17.4% are at risk of developing signs of burnout.

## 5. Methodological Considerations

Because of the cross-sectional design of our study, we cannot make any statistical causal inference. For example, even though we found a positive correlation between low self-esteem and burnout, it does not necessarily mean that burnout boosts low self-esteem or vice versa. In addition, due to lack of identification numbers for the total study population (all social workers at CSIP), we could not compare the background information between respondents and non-respondents. Nonetheless, of the 500 social workers we approached at CSIP, 390 participated in the study, giving a response rate of 78%.

## 6. Conclusion and Recommendations

Burnout found to be more of a risk among social workers who have experienced violence and those with low self-esteem. Therefore, to increase social workers’ well-being, institutions are advised to pay more attention to come up with solutions to prevent the incidence of violence and if prevention fails, immediate respond to physical and mental ailments after violent incidents. In Iranian organizational setting little attention is paid to provide a safe environment and guidelines in office settings (such as physical environment and the arrangement of the visiting room) and field/community settings (such as using a buddy system when making home visits, avoid arguments with family members or lengthy emotional conversations). Rather, recommendations mainly concentrate on educating methods of establishment empathy and good communication skills in working with clients in social work literature of Iran (Chelak, 2009). This approach leads social workers to acknowledge emotional responses to threat and violence, reduced feeling of self-worth and eventually increased risk of burnout. Training alone cannot protect social workers without proper institutional support. Supervisors and managers of social work institutions must take social workers’ concerns seriously and take responsibility for providing an environment that encourages open discussions about violence without feeling of stigma or fear of retribution.

Attention is needed to provide a safe environment, precautions, and guidelines such as physical environment, evaluations of room furnishing, establishing of methods for calling for help, and having the interview rooms in a safe location while working with clients. Regarding low self-esteem (which was found another indicator of burnout symptoms in our study), we recommend CSIP managers to make additional effort to start interventions with the aim of giving social workers sufficient authority to pursue the work, sufficient financial rewards, equal workload or pay and opportunities for higher education in client related programs.

We suggest agency administrators and leadership/managers of social work units to establish guidelines which acknowledge recognition that client violence is a realistic and legitimate practice concern. Policy documents need to be created in which all accidents of violence and threats are unacceptable and psychological consultancies are needed for a victimized social worker. We must teach social workers to accept that it is not a shame to ask for help. A trustful relationship between staff and managers facilitates reporting and tracking all incidents of violence which in turn increases a sense of belonging, being appreciated and self-worth. CSIP organizational board needs to remember that individual-oriented approaches (e.g., taking break from the job and learning deep relaxation) are relatively insufficient in the workplace where a person has much less control over difficult situations at work or experience lack of fairness.
